# Simultaneous Alcohol and Marijuana Use Among Young Adults: A Scoping Review of Prevalence, Patterns, Psychosocial Correlates, and Consequences

**DOI:** 10.35946/arcr.v42.1.08

**Published:** 2022-04-28

**Authors:** Christine M. Lee, Brian H. Calhoun, Devon Alisa Abdallah, Jessica A. Blayney, Nicole R. Schultz, Meg Brunner, Megan E. Patrick

**Affiliations:** 1 Center for the Study of Health and Risk Behaviors, Department of Psychiatry and Behavioral Sciences, University of Washington School of Medicine, Seattle, Washington; 2 Addictions, Drug, and Alcohol Institute, Department of Psychiatry and Behavioral Sciences, University of Washington School of Medicine, Seattle, Washington; 3 Institute for Social Research, University of Michigan, Ann Arbor, Michigan

**Keywords:** alcohol, marijuana, cannabis, co-use, simultaneous, review, young adult

## Abstract

**BACKGROUND:**

Alcohol and marijuana are commonly used by young adults, and use of both substances, particularly at the same time, is prevalent among this population. Understanding the prevalence, patterns, correlates, and consequences of simultaneous alcohol and marijuana (SAM) use is important to inform interventions. However, this literature is complicated by myriad terms used to describe SAM use, including use with overlapping effects and same-day co-use.

**OBJECTIVES:**

This scoping review identifies and describes the peer-reviewed literature focused on SAM use by young adults and distinguishes simultaneous use from same-day co-use of alcohol and marijuana. This review also provides a narrative summary of the prevalence of SAM use, patterns of SAM and other substance use, psychosocial correlates, and consequences of SAM use.

**ELIGIBILITY CRITERIA:**

This review is limited to papers written in English and published in peer-reviewed journals between January 2000 and August 2021. It includes papers assessing simultaneous use or same-day co-use of alcohol and marijuana among young adults ages 18 to 30. Review papers, qualitative interviews, experimental lab studies, policy work, toxicology or medical reports, and papers focused on neurological outcomes are excluded.

**SOURCES OF EVIDENCE:**

PubMed, PsycINFO, and Web of Science databases were searched. Databases were selected and the search strategy developed in consultation with an information specialist.

**CHARTING METHODS:**

A data charting form was utilized to specify which information would be extracted from included papers. Eight categories of data were extracted: (1) research questions and hypotheses; (2) sample characteristics; (3) study procedures; (4) definition of SAM use; (5) prevalence of SAM use; (6) patterns of SAM and other substance use; (7) psychosocial correlates of SAM use; and (8) consequences of SAM use.

**RESULTS:**

A total of 1,282 papers were identified through initial search terms. Through double-blind title/abstract screening and full-text review, the review was narrowed to 74 papers that met review inclusion criteria. Review of these papers demonstrated that SAM use was prevalent among young adults, particularly among those who reported heavier quantities and more frequent use of alcohol and marijuana. Enhancement-related motives for use were consistently positively associated with SAM use. SAM use was associated with greater perceived positive and negative consequences of alcohol and/or marijuana use. Inconsistencies in prevalence, patterns, correlates, and consequences were found between studies, which may be due to large variations in measurement of SAM use, populations studied, methodological design (e.g., cross-sectional vs. intensive longitudinal), and the covariates included in models.

**CONCLUSIONS:**

The literature on simultaneous use and same-day co-use of alcohol and marijuana has expanded rapidly. Of the 74 included papers (61 on SAM use; 13 on same-day co-use), 60 papers (47 on SAM use; 13 on same-day co-use) were published within the last 5 years. Future research focusing on the ways in which SAM use confers acute risk, above and beyond the risks associated with separate consumption of alcohol and marijuana, is needed for understanding potential targets for intervention.

Alcohol and marijuana are two of the most commonly used substances among young adults in the United States. In the past year, approximately 82% of young adults ages 19 to 30 reported alcohol use and 42% reported marijuana use.^1^ Independently, these two substances are associated with numerous short- and long-term risks and harms.^2^–^5^ Those who use both alcohol and marijuana, and in particular those who use both at the same time so that the effects overlap, experience more negative consequences (e.g., getting hurt, heated arguments, trouble with the law) than do individuals who use the substances separately (e.g., alcohol-only or marijuana-only use) or use on the same day but their effects do not overlap.^6^,^7^ Furthermore, cannabis use disorder and alcohol use disorder often overlap, with more than 86% of individuals with a history of cannabis use disorder also meeting current criteria for alcohol use disorder.^8^,^9^ Thus, understanding alcohol and marijuana use—and more specifically simultaneous use of these substances—is critical for the development of prevention and intervention efforts aimed at reducing consequences during the high-risk developmental period of young adulthood.

Simultaneous alcohol and marijuana (SAM) use is generally defined as using both substances at the same time so that their effects overlap. However, this terminology is not always consistent, and SAM use is sometimes also referred to as same-day use, co-use, or cross-fading, among other terms. In contrast, use of both alcohol and marijuana in general, but not necessarily at the same time or on the same day, is considered concurrent use; this is also sometimes referred to as co-use, polysubstance use, or co-occurring use, among other labels.^7^,^10^ A recent focus in the literature has been on trends in concurrent use, such as how changes in marijuana use are associated with changes in alcohol use, and whether use of the two substances is based on complementary (i.e., rising and falling together) or substitution (i.e., one replaces use of the other) effects. (For reviews, see Guttmannova et al.,^11^ Subbaraman,^12^ and Risso et al.^13^) Given the variation in the operationalization of SAM use, and the application of often similar or the same terms to SAM use as concurrent use, it can be difficult to synthesize the literature specific to SAM use. Not only is it important to understand associations between alcohol and marijuana use in general, or among people who use both, but there is a need to better understand the prevalence, patterns, correlates, and consequences associated with simultaneous use. This is particularly important among young adults, as SAM use prevalence among this age group has been increasing historically.^14^ Recent data suggest that many who use both alcohol and marijuana sometimes use both simultaneously^6^,^15^ and are at the highest risk for engaging in SAM use.^14^,^16^

Recent acknowledgment of the need to identify situational risk factors has led to the examination of proximal predictors of SAM use, including social contexts. The use of timeline follow-back (an assessment method using a calendar and anchoring dates to obtain substance use estimates with retrospective reports on each day of a given period),^17^ and daily and ecological momentary assessments (i.e., repeated assessments of substance use behaviors in real time and natural environments)^18^ have provided a finer-grained understanding of patterns, correlates, and consequences at the event level. These repeated-measures methods allow for examination of associations between people (e.g., what distinguishes individuals who engage in SAM use from those who do not) and within people (e.g., what distinguishes situations when SAM use occurs compared to when it does not).

## The Current Study

The purpose of the present scoping review was to do a comprehensive search for papers referencing SAM use by young adults and to organize the authors’ current understanding around this literature to inform future research and intervention work. To the authors’ knowledge, this is the first scoping review of this kind. Given the variability in definitions of SAM use in the extant literature, this review was inclusive of studies that examined use of both alcohol and marijuana on the same day without specifying use at the same time or within a specified time period (i.e., same-day co-use), to allow for greater synthesis of findings across study populations and research designs as well as for comparison of SAM use and same-day co-use. The objective of this review was to summarize research on the prevalence of SAM use, patterns of SAM and other substance use, psychosocial correlates (i.e., motives, norms, situational contexts), and consequences of SAM use. Where appropriate, results from studies utilizing repeated-measures designs to summarize the field’s current understanding of situation-level risk are highlighted.

## Methods

### Protocol and Registration

The protocol was based on the 22-item Preferred Reporting Items for Systematic Reviews and Meta-Analyses extension for Scoping Reviews (PRISMA-ScR).^19^ The protocol was not preregistered, but it can be obtained upon request from the corresponding author.

### Eligibility Criteria

Sources of evidence (i.e., papers) were eligible for inclusion if they (1) were published in peer-reviewed journals between January 2000 and August 2021, (2) were written in English, (3) used human participants in the young adult age range (e.g., ages 18 to 30), and (4) included a focus on or measurement of simultaneous use or same-day co-use of alcohol and marijuana. Papers were excluded if they were review papers, experimental laboratory research, qualitative research, or if they exclusively evaluated policy. In addition, the criteria were refined to exclude neuroscience studies (however, one was included that discussed patterns of SAM and other substance use) and those in which SAM use was based on toxicology or medical reports. The young adult age-related inclusion criterion was meeting one or more of the following: (1) the majority (51% or more) of the sample was between the ages of 18 and 30; (2) the mean or median age of the sample was between the ages of 18 and 30; (3) participants were in 12th grade or college (even if the age was not provided); or (4) an age range that included ages outside of 18 to 30, but with separate findings provided for young adults ages 18 to 30.

### Information Sources and Search Strategy

**Figure 1:**
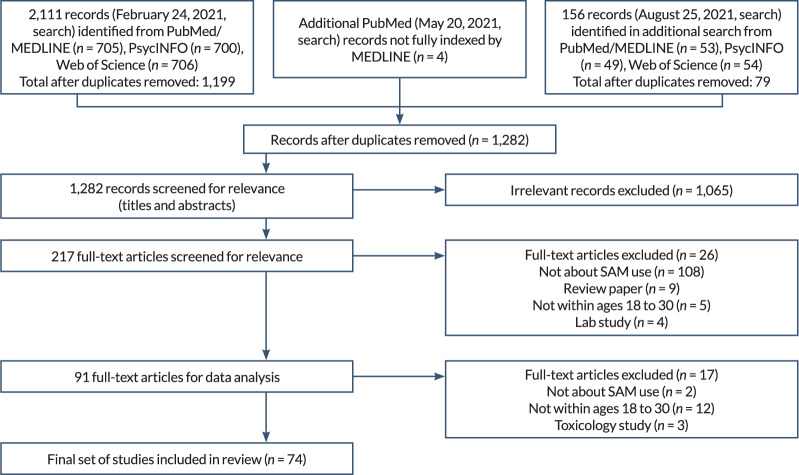
Flow diagram showing literature search and selection of articles. *Note:* SAM, simultaneous alcohol and marijuana.

Electronic databases searched included PubMed, PsycINFO, and Web of Science. The electronic search strategy was developed by the team’s information specialist and refined through team discussion (see Table 1). The initial search was performed on February 24, 2021. After removing duplicates, papers identified by the search were entered into a Covidence database, which facilitates the use of PRISMA methodology (see Figure 1). An additional PubMed search without the MEDLINE-limiter “humans” was performed on May 20, 2021, to screen papers included in PubMed but not indexed by MEDLINE (e.g., smaller journals, manuscripts deposited into PubMed Central); a final search was conducted on August 25, 2021, to update search results prior to publication. These additional searches used the same strategy as the initial search and were performed by the team’s information specialist.

### Selection of Sources of Evidence

Sources of evidence were selected through double-blinded title and abstract screening and full-text review performed in Covidence by four of the authors. The titles and abstracts of all papers identified by the electronic database search were screened by two of the four authors involved at this stage to assess eligibility for inclusion. The full texts of papers not excluded during title and abstract screening were also reviewed by two of the four authors to definitively determine whether papers met all eligibility criteria. Reasons for exclusion decisions were catalogued by Covidence, and disagreements were resolved through discussion.

### Data Charting Process and Data Items

Prior to data extraction/charting, the research team developed a data charting form specifying which information would be extracted from included papers. Eight categories of data were extracted: (1) research questions and hypotheses; (2) sample characteristics (i.e., eligibility criteria, age, gender, race/ethnicity) and recruitment procedures; (3) study procedures (i.e., study design, analytic method); (4) SAM use definition; (5) prevalence of SAM use; (6) patterns of SAM and other substance use; (7) psychosocial correlates of SAM use; and (8) consequences of SAM use. Findings generally were extracted only from the text of the results sections to limit assumptions in interpretations of these findings. Information included in tables but not described in the text of the results sections was generally not extracted. The authors met several times to discuss what types of information were to be collected in each category. Papers were divided among the authors, who then extracted the relevant data into the data charting form for each paper. Data items and categories were then divided among authors, and a second author reviewed and revised the extracted data in the data charting form for each data item/category.

### Synthesis of Results

Evidence from included papers was grouped into the four areas identified in the review’s objectives: (1) prevalence of SAM use, (2) patterns of SAM and other substance use, (3) psychosocial correlates, and (4) consequences of SAM use. Results are presented in narrative format. Some papers provided evidence in more than one area of focus and are included in more than one subsection of the results. Other papers that did not clearly specify SAM use (e.g., those that assessed a broader range of polysubstance use that included illicit drugs such as cocaine, 3,4-methylenedioxymethamphetamine (MDMA or Ecstasy), or psilocybin mushrooms in addition to alcohol and marijuana) or did not directly test associations within the review’s objectives (e.g., papers in which SAM use was tested as a moderator) are retained in Appendix 1 but are not described in the Results section.

## Results

### Selection of Sources of Evidence

As shown in the PRISMA diagram in Figure 1, the initial electronic database searches conducted in February 2021 identified 2,111 records (1,199 nonduplicate papers) related to SAM use or same-day co-use that were written in English and published in peer-reviewed journals between January 2000 and February 2021. After abstract and title screening, 179 papers were deemed eligible for full-text review. After full-text review, 55 papers met all inclusion criteria and were included in the scoping review. A second PubMed search was conducted in May 2021 yielding four additional records (no duplicated papers), all of which were deemed eligible for full-text review and three of which are included in the scoping review. A third search of all three databases in August 2021 identified 156 records (79 nonduplicate papers) published since the date of the initial search (February 2021), of which 34 were deemed eligible for full-text review and 16 met all inclusion criteria and are included in the scoping review. In summary, 1,282 nonduplicate papers related to SAM use or same-day co-use were identified, 217 papers underwent full-text review, and a total of 74 papers are included in this scoping review.

### Characteristics of Sources of Evidence

Appendix 1 provides a list of all 74 papers identified in the final search for relevance for this scoping review. The appendix includes each paper’s methodological design, population, age range, sample size, SAM definition, and whether it is included in the Results section of this review in reference to prevalence, patterns, correlates, and/or consequences of SAM use.

To capture all relevant papers, the authors started the search with inclusive terms for young adult and concurrent or simultaneous alcohol and marijuana use and then systematically reviewed these papers for relevance to SAM use or same-day co-use. This process resulted in a set of papers that was more focused, but continued to vary widely in sample, methods, and measures. The time frames (e.g., yesterday, past month, past 3 months, past year) and response options (e.g., dichotomous, ordinal) of SAM use measures differed between papers. Of the papers included in this review, use was operationalized into four categories based on whether alcohol and marijuana use were specified as occurring simultaneously or overlapping or within different dimensions of same-day use. The categories include using alcohol and marijuana:

- At the same time or together so that their effects overlapped (*n* = 27 papers)
- On the same day within a specified time period (e.g., within 3 hours of each other; *n* = 9 papers)
- At the same time or together without specifying that their effects overlapped or at the same event or occasion without specifying overlapping effects of use within a specified time period (e.g., at the last party attended, during the current night out; *n* = 25 papers)
- On the same day without specifying that they were used together or within a specified time period (*n* = 13 papers)

After careful discussion, the authors categorized SAM use as being inclusive of the first three categories. The fourth category was considered “same-day co-use”—rather than SAM use—because it could not be determined whether alcohol and marijuana use were overlapping or used in relatively close timing with each other. The same-day co-use category was included in this review given varying definitions of SAM use to sometimes include these types of definitions. By inclusion, it may help specify differences in findings. Therefore, of the 74 included papers, 61 were categorized as SAM use and 13 as same-day co-use.

Of the 74 papers, 36 analyzed cross-sectional data and 38 analyzed longitudinal data. Of the papers reporting longitudinal data, nine used data from panel studies with various follow-up intervals, and 22 used data from daily or ecological momentary assessment studies that allowed for testing between- and within-person associations. The remaining seven papers used data collected via the timeline follow-back method, in which participants reported their substance use at a single time point, but the assessment referenced a past series of days (e.g., past month), resulting in a series of day- or occasion-level substance use reports.

Of the 74 included papers, 45 (61%) focused exclusively on young adults ages 18 to 30; 18 (24%) used samples including individuals on the younger end of the age range (e.g., 12th-grade students) or included both late adolescents and young adults; and 11 (15%) included a larger age range of adults, with either a majority of the sample in the young adult age group or estimates stratified by age ranges.

### Prevalence of SAM Use

There were eight papers from nationally representative U.S. samples. Six were from the Monitoring the Future (MTF) study, and two were from the National Alcohol Survey. Estimates based on MTF data indicated that 20% to 25% of 12th-grade students (modal age 18) reported past-year SAM use, both when averaging across longer time periods (e.g., 1976–2011) and shorter, more recent periods (e.g., 2007–2016).^15^,^20^–^22^ An estimated 6% to 7% of 12th-grade students engaged in SAM use most or all of the time.^20^,^21^ Similar findings were noted at later ages (e.g., modal ages 19 or 20 through 29 or 30) in papers following MTF participants longitudinally.^14^,^16^ Estimates based on National Alcohol Survey data found that approximately 15% of young adults ages 18 to 29 who reported drinking in the past year also reported past-year SAM use in data from 2000, 2005, and 2010.^6^,^23^

#### Historical trends

Three papers, all from MTF, reported on historical trends in SAM use over sufficiently long periods of time with nationally representative U.S. samples.^14^,^20^,^21^ Overall trends in SAM use were closely tied to trends in marijuana use and alcohol use.^14^,^20^,^21^ Among 12th-grade students who reported marijuana use, SAM use trends were highly correlated with alcohol use.^21^ Correspondingly, among young adults who reported alcohol use, SAM use trends were highly correlated with trends in marijuana use prevalence.^14^ Generally, the prevalence of past-year SAM use among 12th-grade students was highest in the late 1970s, decreased throughout the 1980s and early 1990s, increased during the mid- and late 1990s, and was relatively stable from the late 1990s until 2007, when a slight increase was observed through 2011.^20^ Among young adults who used alcohol, SAM use trends varied by age.^14^ For those ages 19 to 28, SAM use prevalence generally decreased from the mid-1970s through the early to mid-1990s, but prevalence was stable for those ages 29 or 30.^14^ From the early to mid-1990s through 2011, trends continued to vary by age, ranging from an increase through the mid-2000s followed by no significant change for those ages 19 or 20, to generally consistent increases in use for those ages 21 to 26, to stable use prevalence for those ages 27 or 28.^14^

#### Demographic characteristics

Most papers examining gender and/or sex differences in SAM use, including those using nonrepresentative samples, found that a greater proportion of males than females engaged in SAM use.^15^,^23^–^28^ One paper also found that males consumed greater amounts of alcohol and were high for greater lengths of time on SAM use days than females.^29^ Fewer papers examined race/ethnicity differences. Those that did generally found that White young adults, in comparison to young adults of other racial/ethnic groups, were more likely to engage in SAM use, did so more frequently, and tended to consume greater quantities of alcohol and marijuana when engaging in SAM use.^15^,^16^,^21^ However, these findings were not consistent, and some depended on whether analyses were bivariate or multivariate. Only one paper examined age differences in SAM use during young adulthood with rigor.^14^ This paper used MTF data to estimate SAM use prevalence among young adults who drank alcohol at six modal ages and found SAM use prevalence was highest between ages 19 and 22 at approximately 30%, decreased throughout the twenties, and reached 19% at modal age 29 or 30. A few papers examined differences in SAM use between full-time 4-year college students and non–college students.^16^,^30^ One paper found the likelihood of SAM use was higher for college students not living with their parents relative to those living with their parents.^16^ Another paper found that the within-person association between alcohol and marijuana use was weaker for college students compared to young adults not in college.^30^

### Patterns of SAM and Other Substance Use

SAM use appears to be most common among individuals who use alcohol, marijuana, or illicit drugs more frequently and in greater amounts. Many papers found SAM use was greater among those who engage in heavier drinking and marijuana use.^16^,^20^–^24^,^28^,^31^–^38^ For instance, one paper found that SAM use was most prevalent among those using four or more modes of cannabis administration (e.g., joint, bong, vape, edibles).^39^ Another found that individuals who engaged in more frequent SAM use had a greater likelihood of any illicit drug use (not including marijuana).^21^

Six papers using mixture models (e.g., latent class/profile analysis) to examine patterns of SAM use with other substance use found similar results. Generally, latent classes with high probabilities of SAM use also had high probabilities of other risky substance use behaviors (e.g., using alcohol and marijuana with greater frequency or in greater quantities, experimentation with illicit drugs).^15^,^40^–^42^ In three of these papers, SAM use distinguished one or more latent classes of individuals who use substances from others.^15^,^40^,^41^ The probability of using tobacco and other drugs (i.e., other than alcohol, marijuana, tobacco) was 50% or greater in each profile associated with SAM use.^43^ One paper using mixture models was an exception in that it found that the latent class with the lowest probabilities of substance use reported the highest past-year frequencies of SAM use.^44^ However, this paper’s findings may be biased due to its eligibility criteria (e.g., past-year alcohol, marijuana, and tobacco use), sampling method (i.e., convenience sampling from Craigslist), and sample characteristics (i.e., 89% male; 86% White).

Papers examining daily associations of SAM use or same-day co-use with alcohol and marijuana in terms of consumption and intoxication have produced inconclusive findings. Regarding daily associations between SAM use and alcohol intake, one paper found that young adults consumed more alcohol on SAM use days relative to alcohol-only use days,^32^ whereas another paper found no differences in alcohol (number of drinks) or marijuana (number of hits) consumption on SAM use days relative to alcohol- and marijuana-only use days, respectively.^45^ For same-day co-use, several papers found that more alcohol was consumed on days marijuana was also used relative to days that only alcohol was used.^46^–^48^ Between-person findings in these papers provided some evidence that greater average alcohol intake was associated with more frequent SAM use^32^ and less frequent same-day co-use.^46^,^47^

Regarding daily associations between SAM use and intoxication, one paper found that young adults reported greater subjective intoxication on SAM use days as compared to both alcohol-only and marijuana-only use days,^49^ whereas another found no differences in level of subjective intoxication on SAM use days as compared to both alcohol-only and marijuana-only use days.^45^ Some evidence suggests that SAM use may moderate associations between alcohol and marijuana intake and subjective intoxication such that these associations are weaker on SAM use days relative to alcohol-only and marijuana-only use days, respectively.^49^ For same-day co-use, one paper found that estimated blood alcohol concentrations were higher on days when both alcohol and marijuana were used relative to days when only alcohol was used.^46^ Another paper examining same-day co-use found that young adults tended to drink less alcohol on days when marijuana was used before alcohol.^50^

### Psychosocial Correlates of SAM Use

#### Situational and peer context

Eight papers examined contexts associated with SAM use.^21^,^25^,^31^,^38^,^51^–^54^ Overall, context was an important correlate associated with SAM use across samples (community, treatment seeking) and designs (cross-sectional, event-level). However, findings on specific settings were mixed. Among papers using cross-sectional data, SAM use was significantly less likely to occur in bars and restaurants compared to outdoor and public locations (e.g., park, beach).^52^ However, the likelihood of SAM use was higher in settings in which more people were perceived to be intoxicated,^52^ and individuals had increased odds of SAM use if they engaged in more alcohol and/or marijuana use in certain settings (e.g., park).^21^ In contrast, among a sample of treatment-seeking adults in Canada, SAM use was more likely than marijuana use alone to occur across settings and social compositions, including at home (alone or with friends), at work/school (alone or with friends), with strangers, at bars or taverns, and when driving a car.^25^

Findings from papers using daily or ecological momentary assessment data were also mixed. Associations between contexts and SAM use seemed to differ based on participants’ ages as well as whether the comparison day was alcohol-only or marijuana-only use.^51^,^54^ One paper found that college students were more likely to engage in SAM use—compared to alcohol-only and marijuana-only use—at a friend’s place.^54^ These students were also more likely to engage in SAM use at parties and less likely to engage in SAM use at a bar or restaurant relative to alcohol use only.^54^ This paper also found that college students were more likely to engage in SAM use relative to marijuana use only in contexts with greater numbers of people.^54^ Another paper found that associations between SAM use and contexts differed between young adults under age 21 and those age 21 and older.^51^ For those under age 21, SAM use was more likely to occur at home than alcohol-only use, but odds of SAM use across other physical contexts did not differ from alcohol-only use. For those age 21 and older, SAM use, compared to alcohol-only use, was more likely to occur at a friend’s house or outdoors and less likely to occur in a bar or restaurant. For those age 21 and older but not those under age 21, SAM use was less likely than alcohol-only use to occur when young adults were alone.^51^

Two papers using longitudinal data examined the relationship between social networks and SAM use. In a paper on the role of friends among college students, data collected over two semesters showed that having a greater proportion of friends who used alcohol or marijuana was related to greater likelihood of simultaneous use compared to concurrent use.^31^ In an investigation of how changes from early to late adolescence were associated with SAM use in young adulthood, time with peers using alcohol and marijuana in sixth or seventh grade was predictive of greater likelihood of SAM use in young adulthood (mean age = 20.7).^53^ Similarly, greater alcohol and marijuana use by a sibling or an important adult during adolescence was associated with SAM use in young adulthood, although family effects were no longer significant when all domains (individual, peer, family, neighborhood) were included.

#### Motives for use

A total of seven papers included measures of motives in relation to SAM use or same-day co-use.^21^,^25^,^55^–^59^ Across samples, designs, and measures, motives (particularly SAM-specific motives) were found to be an important correlate of SAM use. Two papers (one using cross-sectional data and one using longitudinal data) described the factor structure and validity of four-factor SAM-specific motives measures, including motives for conformity, positive effects, calm/coping, and social.^55^,^56^ The subscales from these SAM-specific motives measures were associated with the frequency of SAM use in the past month^55^ and the past 3 months^56^ after controlling for alcohol- and marijuana-specific motives.

Three papers utilized daily methods to assess the associations between motives and SAM use or same-day co-use among community samples.^57^–^59^ In a paper assessing both cross-fading motives (i.e., use of alcohol and marijuana at the same time to enhance the positive effects of alcohol or marijuana) and general substance use motives across SAM use occasions, greater cross-fading motives were associated with alcohol use outcomes at the between- and within-person level.^58^ Further, enhancement, social, and coping motives were positively associated with alcohol and marijuana use at the within-person level, and general enhancement and coping motives were associated with greater alcohol and marijuana use at the between-person level. When examining general or substance-specific motives, elevated enhancement and coping motives on alcohol use occasions and social motives on marijuana use occasions were associated with a greater likelihood of SAM use at the between-person level.^59^ Within-person, elevated conformity, enhancement, and coping motives on alcohol use occasions, as well as social, conformity, and coping motives on marijuana use occasions, were associated with a greater likelihood of SAM use. Finally, compared to days when only marijuana was used, same-day co-use of alcohol and marijuana was associated with elevated marijuana-related enhancement and social motives.^57^ Together, these findings show that enhancement motives emerge as an important correlate of SAM use, but other motives (coping, social, conformity) have mixed findings.

Finally, two papers using cross-sectional data examined the “reasons”^21^ for and “functions”^25^ of SAM use. Similar to the paper on cross-fading motives,^58^ among a national sample of 12th-grade students, using alcohol to increase the effects of another drug had a stronger association with frequency of SAM use than other alcohol-related motives for use.^21^ Finally, compared to marijuana use only, SAM use was more likely to occur across all functions assessed, with the greatest odds occurring for self-medication reasons (e.g., “to calm myself down”) among treatment-seeking individuals in Canada.^25^

#### Social norms

Two papers using cross-sectional data found perceived descriptive norms (e.g., perceptions of prevalence and/or quantity of peer substance use) and SAM use frequency were positively associated in samples of college students^60^ and community young adults.^35^ Further, both papers found that individuals who engaged in SAM use, compared to individuals who used only alcohol^35^ and individuals who used alcohol or marijuana but did not engage in SAM use,^60^ endorsed greater descriptive norms of their friends’ and/or peers’ substance use, as measured by the perceived number of drinks in a typical week^35^ or the percentage of friends and peers who engaged in SAM use at least monthly.^60^

#### Expectancies and perceived risk

Two papers included information related to outcome expectancies for alcohol use^52^ and SAM use.^53^ In one paper, cross-sectional research found that negative expectancies for alcohol-related outcomes were associated with decreased odds of SAM use, but positive expectancies were not associated with odds of SAM use.^52^ SAM-specific expectancies were not assessed. In contrast, a longitudinal study examining changes from early to late adolescence found that increases in positive expectancies of SAM use during late adolescence were predictive of SAM use in young adulthood.^53^

Two papers included perceived risk of SAM use. One paper using daily assessment data from a community sample of young adults found that SAM use was especially likely to occur among those with a lower perceived risk of SAM use.^30^ Another study using cross-sectional data found that individuals who engaged in heavier alcohol and marijuana use were more likely to have experienced cross-fading (i.e., intoxication from alcohol and marijuana at the same time) and perceived cross-fading as more desirable and less risky.^61^

#### Other psychosocial or cognitive factors

A cross-sectional study examining behavioral economic demand indices found that individuals who engaged in SAM use exhibited greater overall expenditures on alcohol compared to individuals who used alcohol and marijuana concurrently; moreover, individuals who engaged in SAM use were less sensitive to alcohol price increases than were individuals who used both substances concurrently.^62^ In additional papers, SAM use was positively associated with sensation seeking among a community sample who engaged in past-year SAM use,^35^ was not associated with working memory in a community sample,^63^ and was less likely to occur on days on which college students used certain adaptive emotion regulation strategies (i.e., reappraisal, problem-solving).^64^ In addition, SAM use was positively associated with depressive symptoms cross-sectionally in a community sample^52^ and in a national sample of young adults.^23^ Compared to alcohol-only use, SAM use and SAM use frequency were associated with higher levels of psychosis, oppositional defiant disorder, and conduct disorder in a community sample of young adults.^28^ Another paper found that young adults who reported more depressive symptoms across 2 years also reported more frequent SAM use; furthermore, during months with more depressive symptoms, young adults engaged in more SAM use compared to months when they used alcohol only (levels of depressive symptoms did not differ across months with SAM use compared to neither alcohol nor marijuana or concurrent use).^65^ Further, SAM use was positively associated with likelihood of alcohol dependence.^23^ Among a Swiss population that engaged in same-day co-use of alcohol and marijuana, symptoms of alcohol use disorder and cannabis use disorder appeared to be associated with distinct clusters of symptoms rather than overlapping disorders.^66^

### Consequences Associated With SAM Use

#### Negative consequences of SAM use

Thirty-three papers (14 cross-sectional, five longitudinal, and 14 event-level) examined associations between SAM use or same-day co-use and the negative consequences of use. The measurement of negative consequences in these papers largely centered around alcohol, and papers varied widely in their definition and measurement of consequences. This assessment typically involved pooling items from existing alcohol and/or marijuana consequence measures and modifying the instructions (e.g., “Below is a list of things that sometimes happen to people either during or after they have been drinking alcohol or using marijuana.”^24^). Among most cross-sectional and longitudinal papers,^6^,^23^,^24^,^28^,^35^,^36^,^38^,^55^,^56^,^60^,^65^,^67^,^68^ evidence consistently suggested a positive association between SAM use or same-day co-use and number of negative consequences experienced, even after controlling for demographics, impulsivity, delinquency, motives, alcohol use, and/or marijuana use. Of these papers, half focused on comparing individuals who engage in SAM use to individuals who use both substances concurrently or individuals who use alcohol only,^6^,^24^,^35^,^36^,^38^,^68^ whereas the remaining focused on SAM use frequency as a predictor of consequences.^23^,^28^,^55^,^56^,^60^,^67^ In both college and community samples, individuals who engaged in SAM use reported a greater number of negative consequences relative to those who used alcohol only,^24^,^35^,^36^ though findings were mixed when comparing individuals who engaged in SAM use with those who used concurrently.^24^,^36^,^38^ Papers on SAM use frequency showed a similar pattern, with more frequent SAM use associated with greater negative consequences.^55^,^56^,^60^

Others have found that using only specific marijuana–alcohol combinations, such as combining only leaf or concentrate marijuana products with beer, during the same occasion may actually decrease the odds of negative SAM-related consequences relative to using multiple marijuana products (e.g., leaf, concentrate, edible) and/or multiple alcohol products (e.g., beer, wine, liquor).^33^ Interestingly, ordering effects (i.e., using alcohol before marijuana vs. using marijuana before alcohol) on same-day co-use occasions were not associated with the number of negative consequences.^49^,^50^ Days with heavy episodic drinking (HED; i.e., 4+/5+ drinks for women/men) and marijuana use were associated with increased risk for consequences relative to days in which young adults engaged in non-HED drinking, non-HED drinking and marijuana use, and/or marijuana-only use.^49^,^69^ Notably, non-HED drinking occasions may not differ from non-HED and marijuana use occasions or marijuana-only occasions with regard to alcohol consequences.^69^

Although most papers examined consequences broadly, a subset of papers investigated specific consequence types, including academic, cognitive, social, sexual, aggression, and sleep-related.^6^,^23^,^24^,^36^,^65^,^67^,^68^,^70^–^72^ Compared to those who used alcohol only, individuals who engaged in SAM use were at higher risk across consequence types,^6^,^23^,^24^,^36^ including alcohol-related harms (e.g., problems with relationships, finances, work, or health).^6^ Fewer papers included individuals who used alcohol and marijuana concurrently but did not engage in SAM use, as a comparison.^6^,^24^,^36^ Among those papers, individuals who engaged in SAM use reported more blackouts, risky driving, and negative academic consequences,^24^,^36^ but differences in social consequences were mixed.^6^,^36^ This elevated risk—both broadly and for specific types of consequences—appeared to be a function of high-intensity drinking (i.e., drinking more than twice the binge drinking threshold)^68^ and more frequent simultaneous use.^24^ Other factors, such as SAM-specific norms and motives, also were found to increase negative consequences,^55^,^56^,^60^,^73^ including those specific to marijuana use^55^ and SAM use.^56^ Interestingly, young adults tended to attribute the consequences they experience more to alcohol use than to SAM use.^24^

Among the papers using daily assessments, both between- and within-person effects of SAM use on negative consequences have emerged.^32^,^33^,^45^,^49^,^58^,^74^,^75^ Although most of the papers in this area assessed consequences specific to substance use type (i.e., alcohol, marijuana, SAM), some combined consequences across substances (e.g., total substance-related consequences).^45^,^49^ At the between-person level, young adults with stronger cross-fading motives on average reported more negative alcohol consequences, but not more negative marijuana consequences.^58^ At the within-person level, the effect of SAM use on negative consequences was more pronounced. Among a sample of youth and young adults, SAM use (relative to alcohol-only use) at the last party attended was associated with greater odds of negative consequences (e.g., getting in a fight, having unprotected sex, experiencing forced sex, getting into a car crash, getting in trouble with parents, having a hangover).^74^ Other papers linked SAM use to greater consequences relative to alcohol-only or marijuana-only use occasions.^45^ Still, not all papers found a link between same-day co-use and consequences after controlling for alcohol and/or marijuana use.^29^,^32^,^67^,^75^ For example, among college men, there was no evidence of same-day co-use increasing the likelihood of interpersonal conflict above and beyond alcohol or marijuana use.^67^

#### SAM use and risky driving

Eleven papers (seven cross-sectional, one longitudinal, and three daily assessment) examined SAM use and risky driving. In these papers, risky driving was typically assessed as a single item (e.g., substance-involved driving, being stopped by the police, tickets/warnings/accidents), with the exception of one community study that incorporated a multiple-item measure of driving risk.^76^ Among college and community samples, individuals who engaged in SAM use were more likely to report risky driving compared to those who used alcohol only,^6^,^20^,^24^,^76^ those who used marijuana only,^76^ or those who co-used alcohol and marijuana.^36^ Relative to individuals who only used marijuana or only drank alcohol, individuals who engaged in SAM use endorsed lower risk perceptions for substance-involved driving.^76^ In a paper on young adults sampled when leaving a college district bar, 45% of participants who engaged in SAM use that night reported intending to drive after leaving the bar relative to 29% of those who used alcohol only.^77^ Findings linking SAM use with a greater likelihood of riding with an intoxicated driver have been mixed, as one paper found evidence supporting this association^78^ and another did not.^34^ A third paper found evidence indicating that same-day co-use was associated with greater odds of riding with an intoxicated driver in comparison to alcohol-only days.^79^

#### Perceived or subjective positive effects or consequences

Four papers using daily assessments explored associations between SAM use and its perceived or subjective positive effects or consequences (e.g., feeling relaxed, social, or buzzed).^29^,^32^,^45^,^58^ Across these papers, the measurement of positive consequences centered around alcohol,^29^,^32^,^58^ marijuana,^29^,^58^ or substance use more broadly.^45^ Findings revealed a positive association between SAM use days and perceived positive consequences of alcohol^32^ and/or substance use,^45^ such that more positive consequences tended to be reported on SAM use days relative to alcohol-only^32^ and marijuana-only days.^45^ Notably, these effects persisted even after controlling for other relevant factors such as demographics, motives, weekend day, alcohol use, and/or marijuana use. A recent paper found no significant differences in average daily counts of perceived positive consequences between planned and unplanned SAM use days.^29^ When considering motives, one paper found that higher cross-fading motives in general were associated with greater perceived positive consequences from alcohol and marijuana; in addition, SAM use days with higher cross-fading motives were associated with greater perceived positive consequences of alcohol.^58^

## Discussion

The search identified 74 papers eligible for inclusion in this scoping review on four broad topics relevant to SAM use and same-day co-use by young adults. The four areas reviewed (i.e., prevalence of SAM use, patterns of SAM and other substance use, psychosocial correlates, and consequences of SAM use) elucidate information relevant for the field.

The literature on young adult SAM use is quickly growing. Of the 74 papers (61 on SAM use, 13 on same-day co-use) included in this review, 60 papers (47 on SAM use; 13 on same-day co-use) were published within the last 5 years (since 2017). However, the number of papers within each topic area was fairly limited, with the exception of consequences. Findings suggest that SAM use is prevalent and associated with negative consequences and perceived positive consequences. Review of the papers using nationally representative samples suggests that up to approximately one-quarter of young adults reported SAM use in the prior year,^15^,^20^–^22^ with a higher prevalence during the transition to young adulthood (i.e., ages 19 to 22).^14^ Two papers indicated 15% of young adults (ages 18 to 29) who drink engage in SAM use;^6^,^23^ however, these two studies were conducted prior to the legalization of nonmedical use of marijuana, which started in 2012 in Washington and Colorado and extended to at least 18 states and the District of Columbia by 2021. More recent findings from nationally representative samples suggest that marijuana use and concurrent use of alcohol and marijuana have been increasing steadily.^10^ Continued investigation of SAM prevalence in representative samples with data post-2012 is needed, including examination of longitudinal time trends. Although this review focuses on trends from representative samples, individual papers often report higher rates of SAM use when the samples are more specific to those who use alcohol and/or marijuana; one paper found that almost 75% of college students who reported past-year use of alcohol and marijuana engaged in SAM use in the past year,^60^ further demonstrating SAM use as a high-risk and prevalent behavior.

There is strong evidence across numerous papers to suggest that engaging in SAM use is common among individuals who engage in heavier and more frequent alcohol and marijuana use, including those who also use illicit substances.^16^,^20^–^24^,^28^,^31^–^38^ Findings from papers with different designs and analytic techniques consistently show that patterns of alcohol, marijuana, and other substance use distinguish those who engage in SAM use from other patterns of use. However, the evidence is less conclusive regarding the predictors and implications of SAM use for alcohol and marijuana use from event-level studies. The lack of consistent findings at the situation level is likely due, at least in part, to great variation in the eligibility criteria of samples (i.e., based on any use of alcohol, marijuana, or both, or use of either or both at particular levels), differences in the measurement and modeling of SAM use (e.g., comparing SAM days to alcohol-only days, marijuana-only days, or co-use days), and the presence or absence of covariates. Additional research is needed on the types of people and the types of situations that are associated with SAM use and consequences, with particular attention paid to the extent to which findings may or may not be generalizable.

Consistent, strong evidence was found across papers demonstrating associations between SAM use or same-day co-use with negative consequences (typically focused on consequences from alcohol use, but also marijuana or combined substance use),^6^,^23^,^24^,^35^,^36^,^55^,^56^,^60^,^67^ as well as several other papers documenting associations between SAM use or same-day co-use with mental health and driving risks.^6^,^20^,^24^,^36^,^76^ These effects were often present even after controlling for relevant demographics, alcohol use, and/or marijuana use. Most of the papers assessed the number of consequences reported, with little consistency in the measurement of consequences; fewer papers focused on specific harms. To inform interventions, further understanding of the impacts of SAM use on various aspects of functioning is needed as well as how young adults evaluate these consequences.

Only four papers examined perceived positive consequences associated with SAM use, and participants generally reported more positive consequences on SAM use occasions than alcohol-only or marijuana-only occasions.^29^,^32^,^45^,^58^ The theoretical and clinical importance of understanding the perceived positive effects of SAM use may be critical to informing interventions aimed at motivations and expectations related to SAM use. For example, research on alcohol expectancies and consequences has found that young adults perceive some expectancies and consequences as positive or neutral, despite these traditionally being included on measures of negative outcomes (e.g., hangovers).^80^,^81^ There is also emerging evidence that individuals have specific motives for SAM use and that these motives are associated with increased risk of SAM use^58^,^59^,^82^ and negative consequences in daily assessment studies.^58^ Across these papers, enhancement-related motives, including to get cross-faded,^58^ were consistently associated with SAM-related behaviors. Surprisingly, only two papers examined social norms related to SAM use,^35^,^60^ despite the large focus on young adult social norms in the alcohol literature.^83^

The authors identified several considerations in interpreting the findings from this review. First, many of the papers reviewed included nonrepresentative samples; thus, it is important to consider inclusion criteria and sample characteristics across papers (see Appendix 1). Sample selection is important for considering the findings, particularly for daily assessment studies, which often use higher-risk samples currently engaging in SAM use. Second, it is important to consider study design and whether or what comparisons are being made to SAM use (e.g., SAM use vs. alcohol-only, marijuana-only, co-use, or non–substance use occasions), particularly when examining effects or negative consequences resulting from SAM use. The question at hand in these studies is determining whether SAM use effects are “worse” than effects on other use days. Often these studies control for the amount of alcohol and/or marijuana and assume the effect of SAM use is multiplicative. That is, controlling for the amount of use is implicitly testing whether, for example, having seven standard drinks and spending 4 hours high from marijuana leads to greater consequences when this substance use overlaps than if it occurs separately. This analytic design leads to a strict test of the impacts or effects of SAM use, and implicit assumptions of these models often are not discussed. Specifically, although research designs that answer questions about between-person effects are important for determining who may be at risk, the focus on between-person differences does not consider why or when risk for or consequences of SAM use might be greater in an individual’s typical day-to-day experience. Conversely, comparisons from daily assessment studies are less universal because the samples are often highly selective. Together, these findings highlight the need for clarity in the descriptions of measures and methods used and the relative benefits and limitations of study designs.

The authors identified some measurement considerations. First, the majority of papers used a dichotomous indicator of any versus no SAM use, which fails to capture the intensity of use of alcohol and/or marijuana. Future studies should include more nuanced measures of SAM use to model this heterogeneity. It is particularly important to specify how SAM use is operationalized in each study to compare results. For example, SAM use that is defined as alcohol and marijuana use that is overlapping or within the same time frame is different than same-day co-use of alcohol and marijuana; different effects may be observed, and there would be different hypothesized mechanisms for risks. As mentioned in the introduction, the terminology for these behaviors varies across studies, which makes synthesizing results challenging. The authors of this review recommend that all authors clearly define the constructs used in their research, while reserving the use of the “simultaneous alcohol and marijuana (SAM) use” terminology for behavior strictly defined as the use of alcohol and marijuana at the same time so that their effects overlap.

Second, consistent with literature related to marijuana use, most studies in this review did not include measurement of marijuana potency or quantity consumed. Unlike alcohol, there is no standard unit measure of marijuana, which is further complicated by differing delta-9-tetrahydrocannabinol (THC) potency and modes of use. Future research should try to include more consistent and nuanced measurement of marijuana use; in fact, the National Institute on Drug Abuse is recommending that researchers utilize a standard THC unit in human subjects research when applicable.^84^,^85^ Further, papers should be reviewed in light of the context in which the data were collected; for example, increases in THC content over time, particularly in states where nonmedical use of marijuana is legal, may confound issues related to SAM use and effects of use. Future research needs more nuanced models and measurements to assess main and synergistic effects of the two substances, including how variations in SAM use may lead to increasing consequences and ultimately to cannabis use disorder and/or alcohol use disorder. Although other polysubstance use is not reviewed here, some studies did include this and suggest that SAM use is an early indicator of simultaneous use with illicit substances.^42^

### Prevention/Clinical Implications

Given that individuals who engage in SAM use tend to use alcohol and marijuana more heavily and more frequently, prevention efforts aimed at identifying these individuals are greatly needed, particularly during young adulthood. Notably, once individuals who engage in SAM use are identified, it will be important to determine whether current empirically supported strategies for reducing alcohol use (e.g., brief motivational interventions, personalized feedback interventions)^86^ also reduce SAM use. However, there is little evidence that these interventions have a secondary impact on marijuana use,^7^,^87^ although research in this area is limited. Further, it is unclear if stand-alone marijuana interventions (though there are fewer empirically supported stand-alone interventions for young adults compared to alcohol interventions)^88^,^89^ have a secondary effect on alcohol or SAM use. Few interventions for SAM use, particularly for young adults, have been conducted and have yielded limited success.^90^ For example, a motivational intervention focused on emerging adult themes (e.g., identity exploration, instability, self-focus, feeling in-between, a sense of possibilities) had no effect on SAM use days,^91^ while a brief motivational intervention with adults visiting the emergency department showed reductions in SAM use days.^92^ Given these mixed findings, the authors of this review encourage more research, first, to better understand the mechanisms by which SAM use may lead to risk, in order to identify the most appropriate intervention targets. Currently, motives for use (e.g., enhancement, cross-fading) as well as social norms may be good candidates for inclusion in interventions. Young adults may self-select into social groups (e.g., higher proportion of individuals who engage in SAM use) or contexts (e.g., private spaces, outdoor locations) that increase the odds of SAM use. At the situation level, use of protective strategies (e.g., limiting alcohol use before marijuana use, having a designated driver) may help reduce consequences on SAM use occasions, including substance-involved driving.

### Limitations of Review

This review should be read within the context of certain caveats, including search terms, databases used, and the inclusion/exclusion process. There may have been relevant papers that were not initially included, based on the selection of search terms and databases (e.g., reports, unpublished papers), or studies that remain unpublished because of null findings. This review focuses on SAM use during young adulthood due to the high-risk nature of this population. Thus, papers focused solely on adolescents younger than age 18 or adults older than age 30 were excluded. There is a growing body of work focused on unique circumstances of SAM use among adolescents,^93^ and future work should continue to explore SAM use among other populations at risk. Additionally, the initial search may have missed papers that referenced general samples of adults more broadly if their abstracts did not mention the inclusion of young adults. Although all papers were independently reviewed by two authors to reduce bias, there may be instances when conceptualizations or terms identified as not fitting the current definition of SAM use were misinterpreted by both reviewers and thus excluded. Finally, this review focused on papers that included self-reported SAM use, survey research, and psychosocial-related variables, and did not review or report outcomes that were based on toxicology or medical reports; neurological, policy, or economic outcomes; or qualitative results. Such research may provide additional context for understanding SAM use, as well as its predictors and consequences, among young adults.

## Conclusions

There continues to be an increasing research focus on SAM use, with new findings emerging quickly. To date, it is clear that SAM use is prevalent among young adults and is associated with perceived positive and negative consequences. However, much remains to be learned. In particular, the ways in which SAM use confers acute risk—above and beyond the risks associated with separate consumption of alcohol and marijuana—need to be identified. Psychosocial correlates identified so far include motives for SAM use and norms about use. Whether these additional constructs could be added to supplement existing alcohol- or marijuana-focused interventions, or whether new stand-alone SAM interventions are needed, remains to be seen. Increased understanding of the mechanisms by which SAM use leads to negative consequences is needed to design and test the most effective intervention strategies.

## Figures and Tables

**Table 1:** Search Criteria for Each Database

Database	Search Strategy	No. of Results Retrieved

**PubMed**	Original search: February 2021 ((adolesc* OR teen* OR youth* OR “young adult*” OR “young people*” OR “young person*” OR college* OR “high school*” OR “secondary school*” OR “emerging adult*”) AND (alcohol OR drink* OR ethanol) AND (marijuana OR cannabi* OR THC) AND ((cross-fad* OR crossfad*) OR (simultaneous* OR concurr* OR cooccur* OR co-occur* OR co-use*))) AND ((humans[Filter]) AND (English[Filter]))	705
	May 2021 search (without the “humans” limit)	4
	August 2021 search (without the “humans” limit)	53

**PsycINFO**	Original search: February 2021 (cross-fad* OR crossfad* OR simultaneous OR concurr* OR cooccur* OR co-occur* OR co-use*) (alcohol OR drinking OR ethanol) AND (marijuana OR cannabi* OR THC) (adolesc* OR teen* OR youth* OR young adult* OR young people* OR college* OR high school* OR secondary school* OR emerging adult*)	700
	Limits: Human, English, all journals	
	August 2021 search	49

**Web of Science**	Original search: February 2021 TS = (cross-fad* OR crossfad* OR simultaneous OR concurr* OR cooccur* OR co-occur* OR co-use) TS = (alcohol OR drinking OR ethanol) AND ALL = (marijuana OR cannabi* OR THC) TS = (adolesc* OR teen* OR youth* OR young adult* OR young people* OR young person* OR college* OR high school* OR secondary school* OR emerging adult*)	706
	Limits: English	
	August 2021 search	54

*Note:* THC, delta-9-tetrahydrocannabinol; TS, topic search.

**Appendix 1:** Sample Descriptions, Categorization of Simultaneous Alcohol and Marijuana (SAM) Use, and Areas of Focus Related to Narrative Review

Citation Author & Year	Citation Number	Study Design	Population	Age (range or mean)	Sample Size	Categorization of SAM*	Inclusion in Narrative Results			
							Prevalence	Patterns	Correlates	Consequences
Subbaraman & Kerr, 2015	6	Cross-sectional	National sample from National Alcohol Survey (2005 and 2010)	Age group 18–29	8,626	SAM use: Unspecified overlap	✓			✓
Terry-McElrath & Patrick, 2018	14	Longitudinal; Panel	Nationally representative sample of 12th-grade students from Monitoring the Future	NR	11,789	SAM use: Overlapping effects	✓			
Patrick et al., 2018	15	Cross-sectional	Nationally representative sample of 12th-grade students from Monitoring the Future survey; sample limited to cases from 1976 to 2016 that reported past-year alcohol and marijuana use	NR	84,805	SAM use: Overlapping effects	✓	✓		
Patrick et al., 2019	16	Longitudinal; Panel	Nationally representative sample of 12th-grade students from Monitoring the Future who participated in longitudinal follow-up at modal ages 19 or 20 from 2007 to 2016	NR	1,719	SAM use: Overlapping effects	✓	✓		
Terry-McElrath, O’Malley, & Johnston, 2014	20	Cross-sectional	Nationally representative sample of 12th-grade students from Monitoring the Future	NR	72,053	SAM use: Overlapping effects	✓	✓		✓
Terry-McElrath et al., 2013	21	Cross-sectional	Nationally representative sample of 12th-grade students from Monitoring the Future	NR	34,850	SAM use: Overlapping effects	✓	✓	✓	
Patrick et al., 2017	22	Cross-sectional	Nationally representative sample of 12th-grade students from Monitoring the Future	NR	24,203	SAM use: Overlapping effects	✓	✓		
Midanik et al., 2007	23	Cross-sectional	National sample from National Alcohol Survey (1999–2001)	Age group 18–29	4,630	SAM use: Unspecified overlap	✓	✓	✓	✓
Jackson et al., 2020	24	Cross-sectional	College students who reported past-year alcohol and marijuana use	Age group 18–24	1,390	SAM use: Overlapping effects	✓	✓		✓
Pakula, Macdonald, & Stockwell, 2009	25	Cross-sectional	Clients from treatment programs in Canada reporting past-year marijuana or cocaine use	Age group 18–29	499	SAM use: Unspecified overlap	✓		✓	
Subbaraman & Kerr, 2020	26	Cross-sectional	Sample includes six representative surveys of adults in Washington state between January 2014 and October 2016	Age group 18–29	5,492	SAM use: Unspecified overlap	✓			
de Oliveira et al., 2013	27	Cross-sectional	Nationwide sample of Brazilian college students	Age group 18–24	12,544	SAM use: Unspecified overlap	✓			
Thompson et al., 2021	28	Longitudinal; Panel	Community sample of youth in 10-year longitudinal study with biennial surveys; data from time points 5 and 6	Time 5 Ages 20–26 Time 6 Ages 22–29	Time 5 464 Time 6 478	SAM use: Time frame specified	✓	✓	✓	✓
Fairlie et al., 2021	29	Longitudinal; Daily/EMA	Community sample who reported SAM use at least once in past 2 weeks and alcohol use at least three times in past month	Age group 18–25	Baseline 409 Daily SAM 322 Daily unplanned SAM 308	SAM use: Overlapping effects	✓			✓
Yeomans-Maldnado & Patrick, 2015	30	Longitudinal; Daily/EMA	12th-grade students in the Midwest who participated in a baseline survey and completed at least one follow-up wave and daily survey	Follow-up X_age_ = 18.3	89	SAM use: Overlapping effects	✓		✓	
Meisel et al., 2021	31	Longitudinal; Panel	Incoming first-year college students	Age group 17–23	1,294	SAM use: Time frame specified		✓	✓	
Lee et al., 2020	32	Longitudinal; Daily/EMA	Community sample who reported SAM use at least once in past 2 weeks and alcohol use at least three times in past month	Age group 18–25	391	SAM use: Overlapping effects		✓		✓
Stevens et al., 2021	33	Longitudinal; Daily/EMA	College students who reported past-year alcohol and marijuana use and past-month SAM use	Age group 18–24	274	SAM use: Overlapping effects		✓		✓
Sukhawathanakul et al., 2019	34	Longitudinal; Panel	Youth who participated in the biennial Victoria Healthy Youth Survey from 2003 to 2013	Age group 22–28	640	SAM use: Time frame specified		✓		✓
Linden-Carmichael, Stamates, & Lau-Barraco, 2019	35	Cross-sectional	National sample who reported alcohol use in the past month	Age group 18–25	1,017	SAM use: Time frame specified		✓	✓	✓
Cummings et al., 2019	36	Cross-sectional	First-year college students who reported any past 3-month substance use	X_age_ = 18.1	610	SAM use: Unspecified overlap		✓		✓
Collins, Bradizza, & Vincent, 2007	37	Cross-sectional	Community and college sample who reported drinking at least one 40 oz container of malt liquor a week	Age group 18–35	639	SAM use: Unspecified overlap		✓		
Looby et al., 2021	38	Cross-sectional	College students from seven universities across six states	X_age_ = 19.9	4,764	SAM use: Unspecified overlap		✓	✓	✓
Swan, Ferro, & Thompson, 2021	39	Cross-sectional	College students from a university in Canada, restricted to those who used cannabis in the last 6 months	Age group 17–26	368	SAM use: Time frame specified		✓		
Arterberry, Treloar, & McCarthy, 2017	40	Cross-sectional	College students in an introductory psychology class at a large, public university	X_age_ = 19.0	897	SAM use: Unspecified overlap		✓		
Cadigan et al., 2019	41	Cross-sectional	Community sample who drank at least once in the past year and are currently enrolled in a 2- or 4-year institution	Age group 18–23	526	SAM use: Unspecified overlap		✓		
Bailey, Farmer, & Finn, 2019	42	Cross-sectional	Sample recruited for overrepresentation of externalizing problems	Age group 18–30	2,098	SAM use: Unspecified overlap		✓		
Linden-Carmichael & Allen, 2021	43	Cross-sectional	Young adults who reported past-month HED and SAM use	Age group 18–25	522	SAM use: Overlapping effects		✓		
Stamates, Roberts, & Lau-Barraco, 2021	44	Cross-sectional	Community sample who reported past-year alcohol, cannabis, and tobacco use	Age group 18–25	510	SAM use: Time frame specified	✓			
Linden-Carmichael et al., 2020	45	Longitudinal; Daily/EMA	Sample recruited near large, public university who reported past-month SAM use and HED in past 2 weeks	Age group 18–25	154	SAM use: Overlapping effects		✓		✓
Gunn et al., 2018	46	Longitudinal; TLFB	Incoming first-year college students in 2-year longitudinal study who reported at least one episode of alcohol and marijuana use during data collection	Baseline X_age_ = 18.4	488	Same-day co-use		✓		
Metrik et al., 2018	47	Longitudinal; TLFB	Veterans who used alcohol and marijuana on at least 1 day in the 180-day TLFB assessment period	X_age_ = 30.0	127	Same-day co-use		✓		
Ito et al., 2021	48	Longitudinal; TLFB	College students in Colorado during the time period when recreational marijuana was decriminalized then legalized	X_age_ = 18.4	375	Same-day co-use		✓		
Sokolovsky et al., 2020	49	Longitudinal; Daily/EMA	College students who reported past-year alcohol and marijuana use and past-month SAM use	X_age_ = 19.8	341	SAM use: Time frame specified		✓		✓
Gunn et al., 2021	50	Longitudinal; Daily/EMA	College students who reported past-year alcohol and marijuana use and past-month SAM use	Age group 18–24	258	Same-day co-use		✓		✓
Linden-Carmichael, Allen, & Lanza, 2021	51	Longitudinal; Daily/EMA	Sample recruited near large, public university who reported past-month SAM use and HED in past 2 weeks	Age group 18–25	148	SAM use: Overlapping effects			✓	
Lipperman-Kreda et al., 2018	52	Cross-sectional	Youth who participated in a randomized community trial in California	Age group 18–30	1,538	SAM use: Unspecified overlap		✓		
D’Amico et al., 2020	53	Longitudinal; Panel	Youth who originally participated in a substance use prevention program in middle school	Follow-up X_age_ = 20.7	2,429	SAM use: Unspecified overlap		✓		
Gunn et al., 2021	54	Longitudinal; Daily/EMA	College students who reported past-year alcohol and marijuana use and past-month SAM use	Age group 18–24	313	SAM use: Time frame specified		✓		
Patrick, Fairlie, & Lee, 2018	55	Cross-sectional	Community sample who, at recruitment, reported drinking at least once in the past year	X_age_ = 21.4	286	SAM use: Overlapping effects			✓	✓
Conway et al., 2020	56	Longitudinal; Panel	College students who reported past-year alcohol and marijuana use and SAM use	Age group 18–24	Baseline 1,014 Follow-up 904	SAM use: Overlapping effects			✓	✓
Arterberry et al., 2021	57	Longitudinal; Daily/EMA	Emergency department attendees who reported illicit drug use or prescription drug misuse in past 4 weeks	Age group 18–25	97	Same-day co-use			✓	
Patrick et al., 2020	58	Longitudinal; Daily/EMA	Community sample who reported SAM use at least once in past 2 weeks and alcohol use at least three times in past month	Age group 18–25	281	SAM use: Overlapping effects			✓	✓
Patrick et al., 2019	59	Longitudinal; Daily/EMA	Community sample who reported SAM use at least once in past 2 weeks and alcohol use at least three times in past month	Age group 18–25	399	SAM use: Overlapping effects			✓	
White et al., 2019	60	Cross-sectional	College students who reported past-year alcohol and marijuana use	Age group 18–24	1,389	SAM use: Overlapping effects			✓	✓
Patrick & Lee, 2018	61	Cross-sectional	Community sample from Washington; screening survey for longitudinal study on social role transitions and alcohol use	Age group 18–23	807	SAM use: Unspecified overlap			✓	
Ramirez, Cadigan, & Lee, 2020	62	Cross-sectional	Community sample who, at recruitment, reported drinking at least once in past year	X_age_ = 21.9	480	SAM use: Overlapping effects			✓	
Schuster, Mermelstein, & Hedeker, 2016	63	Longitudinal; Daily/EMA	Youth who participated in study on smoking and reported at least one episode of marijuana, tobacco, or alcohol use during 5-year follow-up EMA	Follow-up X_age_ = 21.3	287	SAM use: Unspecified overlap			✓	
Weiss et al., 2017	64	Longitudinal; Daily/EMA	Undergraduate psychology students who reported alcohol use at least twice in the past month	X_age_ = 19.2	1,640	SAM use: Unspecified overlap			✓	
Fleming et al., 2021	65	Longitudinal; Panel	Community sample who, at recruitment, reported drinking at least once in the past year	Age group 18–23	773	SAM use: Overlapping effects			✓	✓
Baggio et al., 2018	66	Longitudinal; Panel	Swiss men recruited from national military recruitment centers who reported SAM use in the past year	Baseline X_age_ = 20.0 Follow-up X_age_ = 21.3	Baseline 1,559 Follow-up 991	Same-day co-use			✓	
Brown, Testa, & Wang, 2018	67	Longitudinal; Daily/EMA	First-year college males from large public university	Age group 18–19	427	SAM use: Time frame specified				✓
Davis et al., 2021	68	Cross-sectional	College student sample; for interactive effects, subset of students who consumed alcohol in past year	X_age_ = 18.4	Prevalence 1,234 Interactive effects 997	SAM use: Unspecified overlap				✓
Mallett et al., 2019	69	Longitudinal; Daily/EMA	Third-year college students from a large, public university who were part of a longitudinal study and reported alcohol and other drug use in the past year	X_age_ = 20.1	451	Same-day co-use				✓
Norman et al., 2019	70	Cross-sectional	Individuals in Australia who went to bars or clubs	Age group 20–27	5,078	SAM use: Unspecified overlap				✓
Graupensperger et al., 2021	71	Longitudinal; Daily/EMA	Community sample who reported SAM use at least once in past 2 weeks and alcohol use at least three times in past month	Age group 18–25	409	SAM use: Overlapping effects				✓
Read et al., 2021	72	Longitudinal; Daily/EMA	Females who were part of a long-term longitudinal study on adolescent substance risk	Age group 21–24	174	Same-day co-use				✓
Stevens et al., 2021	73	Longitudinal; Daily/EMA	College students who reported past-year use of alcohol and marijuana	Age group 18–24	281	SAM use: Overlapping effects				✓
Egan et al., 2019	74	Cross-sectional	Youth who participated in a randomized community trial	Age group 15–20	834	SAM use: Unspecified overlap				✓
Merrill et al., 2019	75	Longitudinal; Daily/EMA	College students who reported weekly HED or experiencing at least one negative alcohol-related consequence in past 2 weeks	Age group 18–20	96	SAM use: Unspecified overlap				✓
Duckworth & Lee, 2019	76	Cross-sectional	Community sample who, at recruitment, reported drinking at least once in the past year; data from Month 18	X_age_ = 22.2	511	SAM use: Overlapping effects				✓
Thombs et al., 2009	77	Cross-sectional	Patrons exiting bars in college bar district	Median age = 21	469	SAM use: Unspecified overlap				✓
Patrick et al., 2021	78	Longitudinal; Daily/EMA	Community sample who reported SAM use at least once in past 2 weeks and alcohol use at least three times in past month	Age group 18–25	408	SAM use: Overlapping effects				✓
Hultgren et al., 2021	79	Longitudinal; TLFB	College students who reported past-year use of alcohol and another substance (e.g., marijuana, nicotine)	X_age_ = 20.1	367	Same-day co-use				✓
Roche et al., 2019	94^†^	Longitudinal; TLFB	Non–treatment-seeking regular drinkers in Los Angeles area	X_age_ = 29.0	179	Same-day co-use				
Barrett, Darredeau, & Pihl, 2006	95^†^	Cross-sectional	College students who reported use of at least two substances in their lifetime	X_age_ = 21.7	149	SAM use: Unspecified overlap				
Licht et al., 2012	96^†^	Cross-sectional	Danish adults who reported lifetime history of at least 15 illicit drug experiences (excluding marijuana) and use of MDMA or hallucinogens at least once in the past year	Age group 18–35	59	SAM use: Unspecified overlap				
Olthuis, Darredeau, & Barrett, 2013	97^†^	Cross-sectional	Community sample from Canada who reported lifetime cannabis use	X_age_ = 26.8	226	SAM use: Unspecified overlap				
Østergaard, Østergaard, & Fletcher, 2016	98^†^	Cross-sectional	Bar or club goers in Denmark and England	Age group 18–35	1,019	SAM use: Unspecified overlap				
Wade et al., 2020	99^†^	Cross-sectional	Community sample in Wisconsin	Age group 16–26	75	Same-day co-use				
Coughlin et al., 2021	100^†^	Longitudinal; TLFB	Community sample who reported risky alcohol use in past 3 months and at least 1 day of alcohol use and 1 day of cannabis use in past 30 days	Age group 16–24	468	Same-day co-use				
Linden-Carmichael et al., 2021	101^†^	Longitudinal; Daily/EMA	Community sample who reported past-month SAM use and past 2-week HED	Age group 18–25	154	SAM use: Overlapping effects				
Daros et al., 2021	102^†^	Longitudinal; TLFB	Community sample of regular cannabis users (at least once per month for 6+ months) in Canada	Age group 19–26	153	Same-day co-use				
Lee, Cadigan, & Patrick, 2017	103^†^	Cross-sectional	Community sample who, at recruitment, reported drinking at least once in the past year	X_age_ = 21.4	315	SAM use: Overlapping effects				

* **Categorization of SAM use.** SAM use: Overlapping effects = At the same time or together so that their effects overlapped; SAM use: Time frame specified = On the same day within a specified time period (e.g., within 3 hours of each other); SAM use: Unspecified overlap = At the same time or together without specifying that their effects overlapped or at the same event or occasion without specifying overlapping effects of use within a specified time period (e.g., at the last party attended, during the current night out); Same-day co-use = On the same day without specifying that they be used together or within a specified time period.

Ten papers were identified in the search process and included through data extraction; however, the focus of each paper was outside the specific topics of the current review, or results related to SAM were mostly descriptive and thus not presented in the narrative synthesis.

*Note.* EMA, ecological momentary assessment; HED, heavy episodic drinking; MDMA (“ecstasy”), 3,4-methylenedioxy-N-methylamphetamine; NR, Not reported; SAM, simultaneous alcohol and marijuana; TLFB, timeline follow-back; X_age_, mean age.
